# Injury to the Extrasellar Portion of the Internal Carotid Artery during Endoscopic Transsphenoidal Surgery: A Case Report

**DOI:** 10.3389/fsurg.2022.895233

**Published:** 2022-05-10

**Authors:** Shinichiro Teramoto, Shigeyuki Tahara, Yasuo Murai, Shun Sato, Yujiro Hattori, Akihide Kondo, Akio Morita

**Affiliations:** ^1^ Department of Neurological Surgery, Nippon Medical School, Tokyo, Japan; ^2^ Department of Neurosurgery, Juntendo University School of Medicine, Tokyo, Japan

**Keywords:** endoscopic transsphenoidal surgery, internal carotid artery injury, extrasellar portion, hemostatic procedure, pituitary and parasellar tumor

## Abstract

**Background:**

Injury to the internal carotid artery (ICA) during endoscopic transsphenoidal surgery (ETSS) is a serious complication with a risk of mortality. ICA injury during ETSS usually occurs during intrasellar manipulations and rarely occurs in the extrasellar portion. Several hemostatic procedures have been proposed for ICA injury in the intrasellar portion, whereas hemostatic methods for ICA injury in the extrasellar portion, where the ICA is surrounded by bone structures, are less well known.

**Case Presentation:**

A 65-year-old man with an incidental pituitary tumor underwent ETSS. The petrous portion of the left ICA was injured during resection of the sphenoid septum connected with left carotid prominence using a cutting forceps. Bleeding was too heavy for simple hemostatic techniques. Hemostasis using a crushed muscle patch was tried unsuccessfully during controlling of the bleeding. Eventually, the injured site of the ICA was covered with cotton patties followed by closing with a vascularized pedicled nasoseptal flap. Cerebral angiography immediately after surgery showed no extravasation from the injured site of the left ICA petrous portion. However, a carotid-cavernous sinus fistula originating from the injured ICA site was detected 7 days after surgery, so the vascular reconstructive surgery combined with left ICA occlusion was performed. The overall postoperative course was uneventful.

**Conclusion:**

We believe that emergency application of the cottonoids may be effective for hemostasis against ICA injury in the extrasellar portion during ETSS, but further vascular reconstruction combined with ICA occlusion on the injured side and removal of the cottonoids would be required.

## Introduction

Endoscopic transsphenoidal surgery (ETSS) has become a generally accepted surgical approach for pituitary and parasellar tumors since the recent improvement in endoscope performance (1). Injury to the internal carotid artery (ICA) accounts for approximately 1% of complications during transsphenoidal surgery but seems to occur less frequently in ETSS than in microscopic transsphenoidal surgery (2–6). The mortality rate of ICA injury is severe at 11.8%, and immediate causes include massive intraoperative bleeding, collapse of the ICA, and rupture of pseudoaneurysm that appeared postoperatively (2, 7). Most cases of ICA injury associated with ETSS occur during intrasellar manipulation, for which several hemostatic procedures have been proposed (8–10). However, hemostatic methods effective for ICA injury in ETSS have not been established because of the restricted surgical corridor. In particular, injury to the extrasellar portion of the ICA under endoscopic control is rarely reported, so effective management is unclear. We report a case of ICA injury in the extrasellar portion during ETSS and discuss the hemostatic management.

## Case Presentation

**Figure 1:**
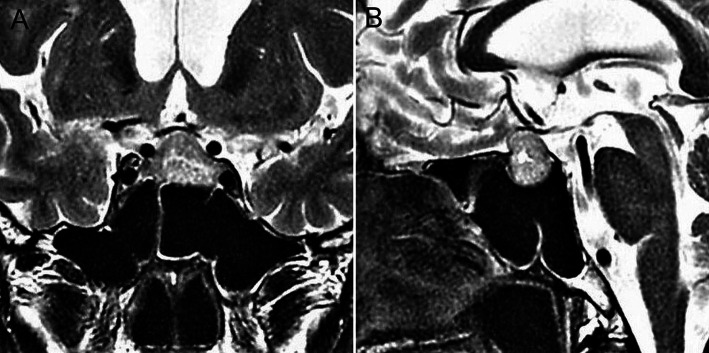
Preoperative coronal (**A**) and sagittal (**B**) T2-weighted magnetic resonance images showing a pituitary tumor as slight hyperintensity, compressing the optic nerve.

A 65-year-old man with a past history of renal cell carcinoma was incidentally found to have a pituitary tumor. The patient had no apparent symptoms indicating visual impairment or pituitary dysfunction and no excessive pituitary hormone levels. Magnetic resonance imaging showed an intrasuprasellar pituitary tumor with a maximum size of 24 mm, appearing as isointensity on T1-weighted imaging, slight hyperintensity on T2-weighted imaging, and compressed the optic nerve assessed as Knosp grade 2 (Figure 1). The sphenoid sinus appeared as the sellar type with no notable findings. ETSS was performed under a relative surgical indication based on tumor compression to the optic nerve and the possibility of metastatic pituitary tumor.

**Figure 2:**
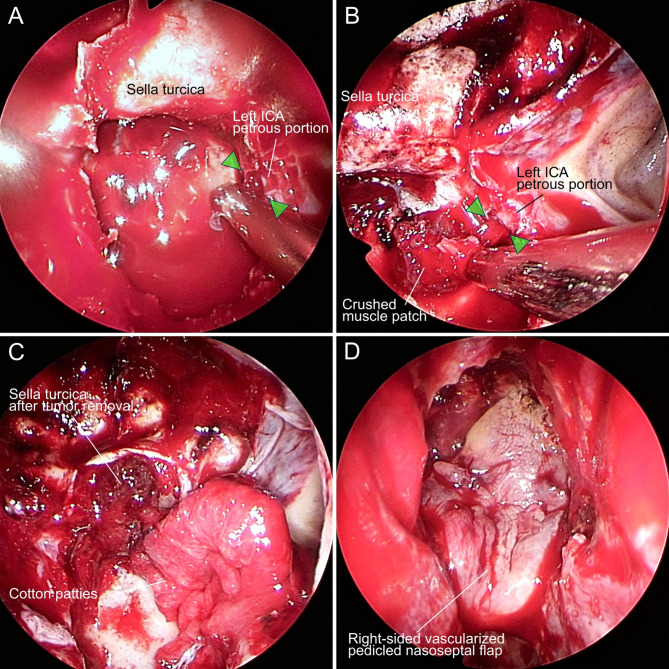
Intraoperative photographs of the endoscopic transsphenoidal surgery that caused internal carotid artery (ICA) injury. (**A**) Petrous portion of the left ICA was injured (green arrowheads). (**B**) Crushed muscle patch was unsuccessfully applied to the injured site (green arrowheads) of the left ICA. (**C**) Injured site of the left ICA was covered with cotton patties. (**D**) Surgical field was closed using the right-sided vascularized pedicled nasoseptal flap.

Sphenoidotomy was performed via a right-sided unilateral paraseptal approach. The sphenoid septum was resected for wide sphenoidotomy to expand the surgical corridor. The petrous portion of the left ICA was injured during resection of the sphenoid septum connected with the left carotid prominence using a cutting forceps (Figure 2A). Cotton patties were immediately placed on the injured site of the left ICA petrous portion to control the massive bleeding. We also asked the anesthesiologist to initiate rapid fluid infusion, order blood transfusions, maintain normal blood pressure, and compress the left cervical carotid artery to relieve the bleeding. The bleeding was suppressed using two suction cannulas of 4 mm and 3 mm diameter. The natural ostium of the left sphenoid sinus was widely opened to allow manipulation through the bilateral nasal cavities. The surgical assistant harvested muscle tissue and fat from the right thigh and prepared muscle tissue in a crushed form. After temporarily stopping the bleeding, a right-sided vascularized pedicled nasoseptal flap was constructed. Application of pressure to the bleeding site with cotton patties was continued, then the dura was incised following the opening of the sellar floor, and the pituitary tumor was resected with suction technique as far as possible. The tumor resection cavity in the sella turcica was filled with fat to prevent cerebrospinal fluid pulsation. We then tried to replace the cotton patties over the bleeding site with the crushed muscle patch (Figure 2B). However, repeated attempts failed to accurately cover the bleeding site with the crushed muscle patch because of the large amount of bleeding caused by removing the cotton patties. We eventually decided not to use the crushed muscle patch and completed the hemostatic procedure by applying two cotton patties to the bleeding site, followed by fibrin glue coating (Figure 2C). The surgical field was closed using the right-sided vascularized pedicled nasoseptal flap with fibrin glue coating (Figure 2D), followed by fixing with balloon compression.

**Figure 3:**
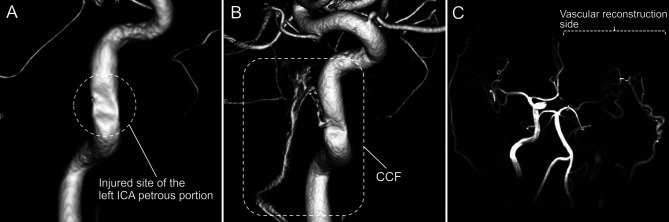
(**A**) Three-dimensional cerebral angiography of the left internal carotid artery (ICA) immediately after surgery showing no extravasation from the injured site of the left ICA petrous portion. (**B**) Three-dimensional cerebral angiography of the left ICA following balloon deflation 7 days after surgery revealing carotid-cavernous sinus fistula (CCF) originating from the injured site of the left ICA petrous portion. (**C**) Postoperative magnetic resonance angiography showing the success of vascular reconstructive surgery combined with internal coil trapping of the left ICA.

Cerebral angiography performed immediately after surgery under general anesthesia showed no extravasation from the injured site of the left ICA petrous portion and normal antegrade cerebral hemodynamics (Figure 3A). The patient was awakened and withdrawn from the respirator after confirming the absence of abnormal findings on brain imaging the day after surgery. He showed good consciousness with no neurological deficit. However, cerebral angiography following balloon deflation 7 days after surgery revealed the presence of a carotid-cavernous sinus fistula (CCF) originating from the injured site of the left ICA petrous portion (Figure 3B). The CCF was believed to result from angiogenesis to repair the injured site of the ICA, which had progressed into the cavernous sinus or ICA laceration extending to the ICA cavernous portion connected to the cavernous sinus. The CCF anterogradely drained into the left inferior petrosal sinus without reflux findings and was asymptomatic. However, we planned to resolve this iatrogenic abnormality.

Superficial temporal artery–middle cerebral artery double bypass followed by complete occlusion of the left ICA due to coil embolization was selected as the treatment plan for the CCF because the patient had cerebral ischemic tolerance in the occlusion test of the left ICA. These procedures were successful, and the postoperative course was uneventful (Figure 3C). The patient was discharged on foot without a neurological deficit. The patient complained only of discomfort in the sense of smell in the postoperative outpatient clinic. Histopathology of the pituitary tumor demonstrated a nonfunctioning pituitary adenoma.

## Discussion

Injury to the ICA during ETSS is rare but likely to be fatal (4–6). The risk factors for ICA injury in transsphenoidal surgery include invasion of the ICA by the tumor, history of similar transsphenoidal surgery, and postradiation therapy to the tumor (2). In the present case, the surgery proceeded despite poor visibility due to blood staining on the endoscope lens, which resulted in the inadvertent ICA injury. Failure to manage such ICA injury can lead to severe anemia, hypovolemic shock, and coagulopathy due to massive blood loss. Therefore, we need to be fully aware of protocols to manage ICA injury under endoscopic control. The proposed intraoperative management for ICA injury is briefly summarized as follows (10, 11):

• Two surgeons adopt a three- or four-hand technique via a two-nostril approach, one to control the blood flow and the other to stop the bleeding site.
• Surgeons use two large-bore suction cannulas and, if available, a foot-controlled endoscope lens cleaning system to maintain a clear surgical field.
• Anesthesiologist administers rapid fluid resuscitation and blood transfusions, maintains normal blood pressure, and infuses heparin to prevent cerebral embolism if necessary.
• Operating room staff and the endovascular surgeon should be informed to prepare for additional treatment after hemostasis.
• Hemostatic methods include applying several hemostatic agents, crushed muscle patch, cottonoids, and fat and interrupting the blood vessel with a vascular clip.

In the present case, we found that the cooperation of two surgeons was efficient against accidental ICA injury, but an around 4-mm diameter suction cannula rather than a larger size would be more suitable for fine hemostatic manipulations. Moving the endoscope lens away from the surgical field and using the zoom function resulted in less frequent staining with blood. Heparin may be used not during the surgery, only if postoperative cerebral angiography detects a thrombus. The presence of at least two anesthesiologists and two operating room staff ensured rapid and flexible support. Repeat cerebral angiography both immediately after surgery and one week after surgery was definitely important, as previously suggested (12).

Various hemostatic methods have been advocated, but the crushed muscle patch is well known to be effective. A crushed muscle patch was emphasized as a more successful hemostatic material than the gelatin-thrombin matrix, oxidized regenerated cellulose, and chitosan hydrogel in an animal model with ICA injury (13), and the clinical efficacy was also reported (14). Placing the crushed muscle patch directly over the ICA injured site without compression promotes the hemostatic action, which later allows formation of a normal vessel wall with adequate patency (13, 14). However, in our case, hemostasis of the ICA injury in the petrous segment could not be achieved by simply applying a crushed muscle patch. The ICA in the extrasellar portion is surrounded by bone structures, which interfered with the tight attachment of the crushed muscle patch to the injured ICA site. In addition, we found maintaining the crushed muscle patch over the ICA injured site and simultaneous blood suctioning was very difficult. In contrast, cotton patties could be applied to the bleeding site with pinpoint accuracy during blood suctioning.

Simultaneous covering of the injured site of blood vessel and blood suctioning is essential for hemostasis, without which overpacking with hemostatic agents is a risk. Application of cottonoids may thus be an advantageous hemostatic method for vascular injury, especially in the extrasellar ICA, where the surrounding bone structures obstruct the accurate application of the hemostatic agents. However, leaving cottonoids in the body always risks triggering infection, cerebral edema, and granuloma formation (15). Therefore, the area of the cottonoids should be closed using a vascularized pedicled nasoseptal flap with blood supply to avoid external access. Furthermore, we believe that vascular reconstruction combined with ICA occlusion on the injured side followed by removal of the cottonoids will be necessary, even if no pseudoaneurysm or arteriovenous fistula develops at the injured ICA site later. We intend to remove the cotton patties in the near future after confirming that the blood flow of the ICA petrous portion is obstructed. Chibbaro et al. had no instances of ICA injury even in difficult cases such as giant pituitary adenoma (16–18). A careful preoperative assessment of tumor characteristics, a multidisciplinary team of Neurosurgery/Otorhinolaryngology specialists, and the assistance of intraoperative navigation and micro-Doppler ultrasonography are beneficial in reducing the risk of unexpected complications in ETSS for pituitary and parasellar tumors, as Chibbaro et al. advocated (16–20).

## Conclusion

Injury to the ICA during ETSS requires immediate and effective complete hemostasis because the prolonged hemostatic effort is directly associated with life-threatening events and cerebral embolism. The present case demonstrated that emergency application of cottonoids to the bleeding site might be effective for the injury to the ICA in the extrasellar portion. However, vascular reconstruction combined with ICA occlusion on the injured side and removing the cottonoids should be considered later.

## Data Availability

The original contributions presented in the study are included in the article/Supplementary Material; further inquiries can be directed to the corresponding author/s.
